# Rapid evolution fuels transcriptional plasticity to ocean acidification

**DOI:** 10.1111/gcb.16119

**Published:** 2022-03-03

**Authors:** Jingliang Kang, Ivan Nagelkerken, Jodie L. Rummer, Riccardo Rodolfo‐Metalpa, Philip L. Munday, Timothy Ravasi, Celia Schunter

**Affiliations:** ^1^ Swire Institute of Marine Science School of Biological Sciences The University of Hong Kong Hong Kong Hong Kong SAR China; ^2^ Southern Seas Ecology Laboratories School of Biological Sciences & The Environment Institute The University of Adelaide Adelaide South Australia Australia; ^3^ Australian Research Council Centre of Excellence for Coral Reef Studies James Cook University Townsville Australia; ^4^ College of Science and Engineering James Cook University Townsville Queensland Australia; ^5^ ENTROPIE – UMR 9220 (CNRS, IRD, UR, UNC, IFREMER) IRD Institut de Recherche pour le Développement Nouméa cedex New Caledonia; ^6^ Marine Climate Change Unit Okinawa Institute of Science and Technology Graduate University Onna‐son Japan; ^7^ State Key Laboratory of Marine Pollution City University of Hong Kong Hong Kong Hong Kong SAR China

**Keywords:** circadian rhythm, climate change, elevated *p*CO_2_, intracellular pH, neuromolecular response, transcriptome

## Abstract

Ocean acidification (OA) is postulated to affect the physiology, behavior, and life‐history of marine species, but potential for acclimation or adaptation to elevated *p*CO_2_ in wild populations remains largely untested. We measured brain transcriptomes of six coral reef fish species at a natural volcanic CO_2_ seep and an adjacent control reef in Papua New Guinea. We show that elevated *p*CO_2_ induced common molecular responses related to circadian rhythm and immune system but different magnitudes of molecular response across the six species. Notably, elevated transcriptional plasticity was associated with core circadian genes affecting the regulation of intracellular pH and neural activity in *Acanthochromis polyacanthus*. Gene expression patterns were reversible in this species as evidenced upon reduction of CO_2_ following a natural storm‐event. Compared with other species, *Ac*. *polyacanthus* has a more rapid evolutionary rate and more positively selected genes in key functions under the influence of elevated CO_2_, thus fueling increased transcriptional plasticity. Our study reveals the basis to variable gene expression changes across species, with some species possessing evolved molecular toolkits to cope with future OA.

## Graphical abstract

Elevated *p*CO_2_ induced common molecular responses related to circadian rhythm and immune system but different magnitudes of response across six species. *Acanthochromis polyacanthus* exhibited many more differentially expressed genes with a more rapid evolutionary rate fuelling the increased transcriptional plasticity

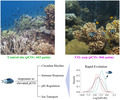

## 1 INTRODUCTION

Global ocean surface pH is projected to decline at a rate of approximately 0.02 pH units per decade with the ongoing uptake of anthropogenic atmospheric CO_2_ by the oceans (Bindoff et al., 2019), a process termed ocean acidification (OA). There is concern as to how marine life will respond and whether adaptation to this rapid acidification is possible. Higher partial pressures of carbon dioxide (*p*CO_2_) in seawater cause blood arterial *p*CO_2_ to increase in fish and other marine animals (Brauner et al., 2019). Fishes can prevent acidosis and maintain their acid–base balance at higher *p*CO_2_ levels by compensatory ion exchange (Brauner et al., 2019), but this can alter energy budgets (Lefevre, 2019) and impact other biochemical processes (Brauner et al., 2019). Therefore, although some fish appear to be relatively unaffected by projected future CO_2_ levels (Clark et al., 2020; Munday et al., 2009b, 2011, 2019a), a decade of laboratory experiments indicate that predicted OA conditions could affect some marine fishes’ physiological performance, growth, survival (Hannan et al., 2020, 2021; Munday et al., 2019a; Nagelkerken et al., 2021), and behaviors (e.g., olfactory, auditory, vision, behavioral lateralization, learning, activity, anxiety, boldness, foraging behavior, and homing ability) (Goldenberg et al., 2018; Jiahuan et al., 2018; Munday et al., 2019a; Nagelkerken & Munday, 2016; Paula et al., 2019a, 2019b; Porteus et al., 2018).

The reported behavioral impairments in elevated *p*CO_2_ conditions have been associated with altered function of GABA_A_ (γ‐aminobutyric acid type A) receptors—the major inhibitory neurotransmitter in the vertebrate brain essential for normal brain function, neuronal activity, information processing, and plasticity through maintaining the polarity and amplitude of Cl^−^ fluxes(Bhat et al., 2010; Heubl et al., 2017; Heuer et al., 2016, 2019; Lai et al., 2015; Nilsson et al., 2012; Schunter et al., 2019). This indicates that these impairments have a neural origin (Heuer et al., 2019; Paula et al., 2019a). Furthermore, other physiological responses to elevated *p*CO_2_, such as ion exchange and acid‐base regulation, are also controlled by the brain (Wang et al., 2020). Consequently, changes in gene expression in the brain caused by elevated *p*CO_2_ can be used to understand the mechanisms underpinning neural effects of elevated *p*CO_2_ in marine organisms and the likely impacts on their behavior, physiology, and adaptive capacity.

In fishes tested in laboratory experiments, gene expression patterns indicate that neural responses to elevated *p*CO_2_ vary considerably among species (Hamilton et al., 2017; Lai et al., 2016, 2017). Nonetheless, some recurring expression changes linked to ion transporters, including the GABA receptors (Schunter et al., 2018; Williams et al., 2019), have been identified from the transcriptional architecture of *Acanthochromis polyacanthus* brains (Schunter et al., 2016, 2018) and *Oncorhynchus kisutch* olfactory tissues (Williams et al., 2019). Notably, *Ac*. *polyacanthus* exhibited a shift in expression of core circadian genes (CCGs) after elevated *p*CO_2_ exposure (Schunter et al., 2016). While these controlled experiments provide valuable insights into the effects of elevated *p*CO_2_ on brain function in fish, they lack the interface with other natural factors such as predators, competitors, water currents, and food supply that are present in the wild (Langdon & Atkinson, 2005; Riebesell & Gattuso, 2015). Therefore, the patterns of gene expression observed in laboratory experiments under elevated *p*CO_2_ may not fully capture the response of fish to future ocean acidification (OA) conditions in nature.

**FIGURE 1:**
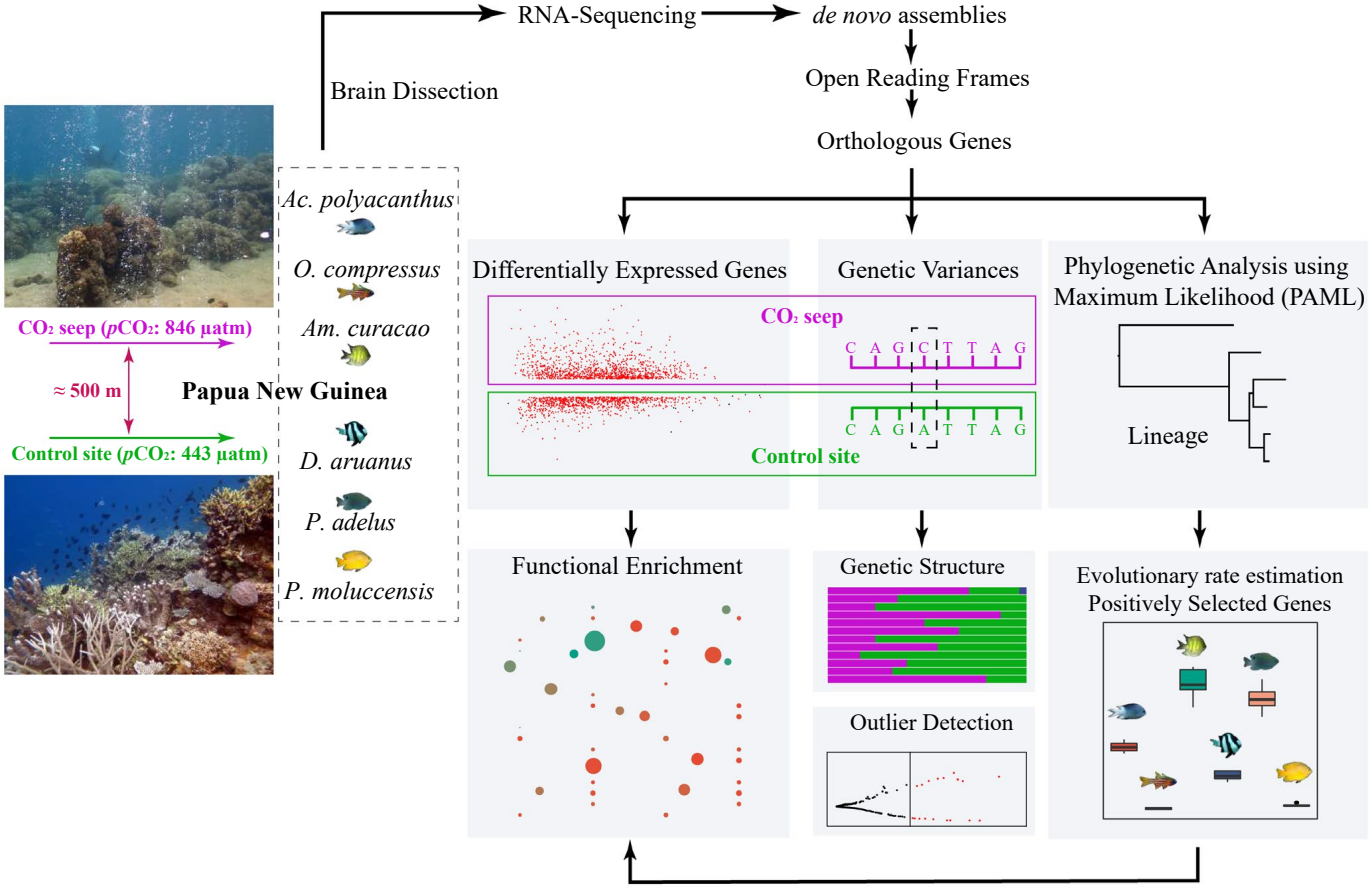
Schematic illustrating samples collected for six species of coral reef fishes and the molecular and bioinformatic analyses pipeline. Wild adult fishes were collected from both the CO_2_ seep (average *p*CO_2_ 846 µatm (Fabricius et al., 2011)) and the adjacent control reef (separated by approximately 500 m, average *p*CO_2_ 443 µatm (Fabricius et al., 2011)), and brains were dissected. RNA extraction and sequencing protocols were performed, and expressed orthologous genes among all species were identified to examine differential gene expression and potential genetic variances between individuals from the CO_2_ seep and control reefs and investigate disparity of protein sequences between species

Volcanic CO_2_ seeps, such as the Upa‐Upasina Reef in Milne Bay Province (Normanby Island, Papua New Guinea), can be used as natural laboratories where CO_2_ rises from the substratum and acidifies the surrounding seawater to levels similar to, or sometimes beyond, the projections for OA by the end of this century (Fabricius et al., 2011; Hall‐Spencer et al., 2008; Munday et al., 2014). These CO_2_ seeps provide a unique opportunity to investigate the longer‐term effects of OA conditions on fishes in their natural habitat and how this varies among species. While previous studies found that elevated *p*CO_2_ at CO_2_ seeps either altered behavior (e.g., reduced predator‐avoidance) and physiology in fishes (Munday et al., 2014; Nagelkerken et al., 2016, 2017), or did not affect predator recognition(Cattano et al., 2017), the direct effect on brain function in wild fishes has not been tested. Here, we sequenced the transcriptomes of the brains of adult fish of five damselfish species (*Ac*. *polyacanthus*, *Amblyglyphidodon curacao*, *Dascyllus aruanus*, *Pomacentrus adelus*, and *Pomacentrus moluccensis*) and one cardinalfish species (*Ostorhinchus compressus*) from a reef within the Upa‐Upasina CO_2_ seep in Papua New Guinea (pH 7.77, *p*CO_2_ 846 µatm (Fabricius et al., 2011)) and an adjacent reef (500 m distance) with ambient *p*CO_2_ (pH 8.01, *p*CO_2_ 443 µatm (Fabricius et al., 2011); Figure 1), including an additional collection of *Ac*. *polyacanthus* individuals from the CO_2_ seep during a storm that flushed ambient *p*CO_2_ water over the CO_2_ seeps site reducing the seep *p*CO_2_. Based on 14,634 orthologous genes across the six fish species, we investigated the effects of natural in situ exposure to elevated *p*CO_2_ by evaluating genetic and brain gene expression variation to detect similarities and disparities in the molecular and functional responses to OA. We further evaluated the evolutionary drivers of adaptive potential in these coral reef fishes to future OA.

## 2 RESULTS AND DISCUSSION

### 2.1 Molecular response across species from the CO_2_ seep

**FIGURE 2:**
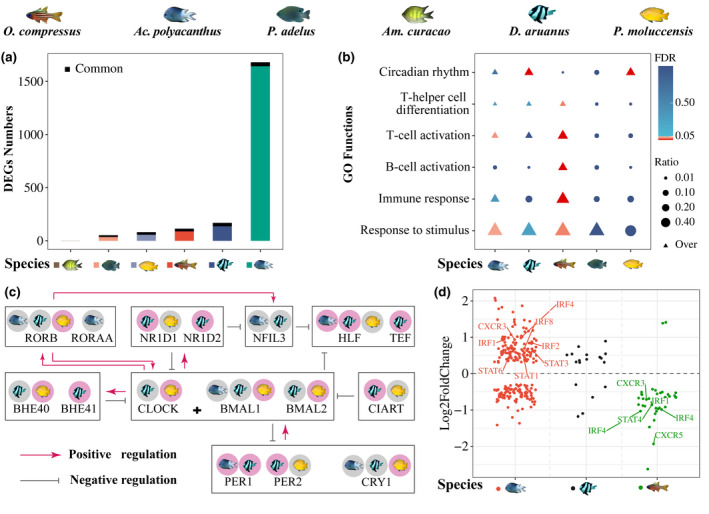
Molecular responses of six coral reef fish species from the natural CO_2_ seep and nearby control reef. (a) number of differentially expressed genes (DEGs) between the CO_2_ seep and control reef for each species. The height of each bar represents the number of DEGs in each species. Each color associated to a fish species indicates the specific DEGs for the species, and the black part shows the overlapping DEGs between this and at least one another species. (b) Functional enrichment of DEGs for each species, the size of the circle or triangle indicates the ratio of DEGs per function to total DEGs per species, the triangle indicates DEGs that were overrepresented in the respective function, red to blue coloration ranged from highly significantly overrepresented to not significant. Three more specific functions (T‐helper cell differentiation, T cell activation, B cell activation) within immune response were displayed separately to show the extraordinarily high proportion of DEGs related to immune response in *O*. *compressus*. (c) Simplified schematic of the molecular transcriptional–translational feedback loops of core circadian DEGs. A red background indicates upregulation and grey indicates downregulation at the CO_2_ seep. Red lines indicate positive regulation, and gray lines indicate negative regulation of the downstream genes. (d) The regulation of immune response DEGs in three species under elevated *p*CO_2_. *Ac*. *polyacanthus* is in red, *D*. *aruanus* is in black, and *O*. *compressus* is in green, with each dot representing one immune response related gene and its upregulation (Log2Foldchange >0) or downregulation (Log2Foldchange <0) at the CO_2_ seep with some key genes highlighted by name

The six coral reef fish species exhibited varying degrees of transcriptional changes in response to elevated *p*CO_2_ (Figure 2a). In fish collected at the CO_2_ seep, compared with the nearby control reef, *Ac*. *polyacanthus* experienced transcriptional changes that were an order of magnitude higher than all other species (1679 differentially expressed genes; DEGs, Table S1), followed by *D*. *aruanus* (169 DEGs, Table S1). In contrast, *Am*. *curacao* only displayed one DEG (C‐X‐C chemokine receptor type 4). Furthermore, no significant genetic sequence variations were detected between individuals of each species collected at the CO_2_ seep and the adjacent control reef (Figure S1a,b). The largest standing genetic variation within species was also observed in *Ac*. *polyacanthus* and *D*. *aruanus* (i.e., 918 and 923 single‐nucleotide polymorphisms [SNPs], respectively; Table S2). The large variations in how the brain of these six coral reef fishes responded to elevated *p*CO_2_ resulted in no genes commonly differentially expressed across all species and a maximum of four DEGs shared among three species. Sixty‐five genes exhibited common differential expression between the CO_2_ seep and control reef for two or more species (Table S3, Figure S2), which were involved in important biological processes, such as the response to stimulus (e.g., HSPB1, HSP47, SUN1), immune response (e.g., IRF1, IRF4, NFIL3), and circadian rhythm (CR) (e.g., PER1, CLOCK, BMAL1) (Figure 2b), indicating the fundamental responses to elevated *p*CO_2_ across species in a natural setting.

CRs are near‐24‐h oscillations found in nearly all aspects of physiological processes in the vertebrate brain and body (Dunlap & Loros, 2016; Logan & McClung, 2019), of which 3–10% of transcripts display rhythmical expression (Staels, 2006). These molecular rhythms are generated by a transcriptional‐translational feedback loop in CCGs (Logan & McClung, 2019). Environmental perturbations can reset CR in vertebrates, which is characterized by changes in expression of CCGs (Logan & McClung, 2019). Elevated *p*CO_2_ exposure has been shown to induce expression changes in the CR pathway in *Ac*. *polyacanthus* (Schunter et al., 2016) as well as an anemonefish, *Amphiprion percula*, in laboratory settings (Schunter et al., 2021). Three species in this study—*D*. *aruanus*, *P*. *moluccensis*, and *Ac*. *polyacanthus*—displayed significant expression differences in CCGs between the CO_2_ seep and control reef (Figure 2c, Table S4). However, these CCGs were modulated differently in *Ac*. *polyacanthus*, with more downstream changes compared with *P*. *moluccensis* and *D*. *aruanus*. The difference is supported by gene interaction networks, which quantify the interaction between the CCGs and other DEGs in the GO term “CR” (GO:0007623) and showed no interaction for *P*. *moluccensis* and *D*. *aruanus* (Figure S3). Of 12 DEGs (Table S4) that directly interacted with the CCGs in *Ac*. *polyacanthus*, some genes regulate the CCGs activity via ubiquitination (UBE3A), phosphorylation (GSK3B), alternative splicing (ANM5), mediating nicotinamide adenine dinucleotide (NAD^+^) biosynthesis (NAMPT), coactivating cAMP response element binding protein (CRTC1), or binding to either the promoter (KLF10) or untranslated region (RBM4). The transcriptional changes of CCGs in *Ac*. *polyacanthus*, therefore, generate a more significant impact on downstream genes. In the main network (1438 genes, 9621 interactions) of all DEGs of *Ac*. *polyacanthus*, 632 and 1307 DEGs are connected to the CCGs by two or three nodes, respectively. This emphasizes the importance of CR in the transcriptional adjustments that occur in the brain in response to elevated *p*CO_2_ and downstream genes are either directly or indirectly changed following the expression shift in CCGs.

An additional differential response between fish from the CO_2_ seep and control reef that was common across the five species and more significant for three species (i.e., *Ac*. *polyacanthus*, *D*. *aruanus*, and *O*. *compressus*) was an immune response (Figure 2b). Intriguingly, *O*. *compressus*, which was the only nocturnal species examined in this study, exhibited an extraordinarily high proportion (38.5%) of DEGs for the regulation of T cell, B cell, and T‐helper cells in fish from the CO_2_ seep compared with the adjacent control reef. In comparison, *Ac*. *polyacanthus* and *D*. *aruanus* exhibited 213 (12.7%) and 18 (10.7%), respectively (Table S5). The expression of nearly all immune genes examined (Figure 2d) were decreased in *O*. *compressus* from the CO_2_ seep, such as interferon regulatory factors (IRF1 and 4), signal transducer and activator of transcription protein (STAT4), and C‐X‐C chemokine receptors (CXCR3 and 5), while *Ac*. *polyacanthus* from the CO_2_ seep exhibited elevated expression of IRFs (IRF1, 2, 4, and 8), STATs (STAT1, 3, and 6), and CXCR3. As such, elevated *p*CO_2_ may have more obvious and adverse effects on immunological responses in *O*. *compressus* compared with *Ac*. *polyacanthus*. Cardinalfishes may be especially sensitive to elevated *p*CO_2_ (Munday et al., 2009a), which may be why *O*. *compressus* induced an elevated immune response. Although we only collected one nocturnal species, the opposing immune response to elevated *p*CO_2_ of *O*. *compressus* might be related to differences in day or night activity, as samples were collected during the day. Moreover, similar opposite trends (i.e., opposite to damselfishes) for *O*. *compressus* were previously observed across various oxygen uptake rate metrics (Hannan et al., 2021). Nonetheless, previous laboratory experiments where fishes were exposed to elevated *p*CO_2_ also indicated a change in the expression of genes involved in the immune response (Machado et al., 2020; de Souza et al., 2014). Hence, immune regulation appears to be an important function involved in the response to elevated *p*CO_2_ across numerous species of reef fishes.

### 2.2 Distinct regulation of intracellular pH and ion transport in *Acanthochromis polyacanthus*

**FIGURE 3:**
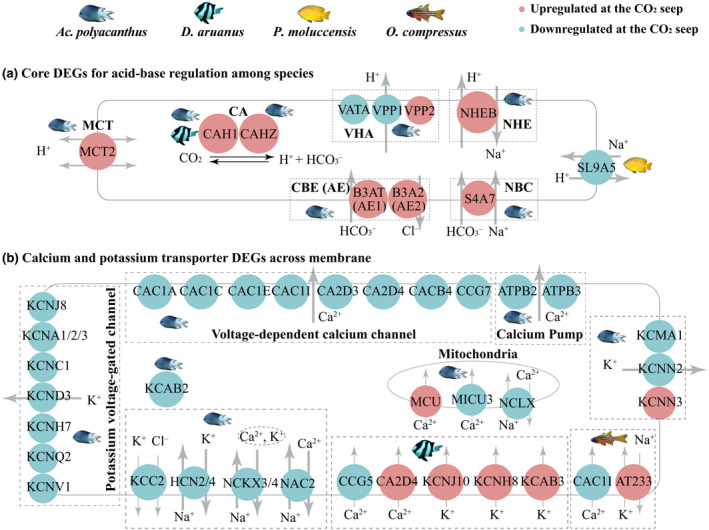
Regulation of pH_i_, Ca^2+^, and K^+^ transport in response to elevated *p*CO_2_ in four coral reef fish species. Genes with significant upregulation at the CO_2_ seep have a red background and downregulation is indicated in blue. (a) Core acid‐extruder and uptake genes involved in acid–base homeostasis with differential expression for the different species. (b) Overall downregulation of calcium and potassium transporters across the cell membrane in *Ac*. *polyacanthus*

A key challenge for fishes when coping with elevated *p*CO_2_ is maintaining blood and tissue pH homeostasis to avoid acidosis. *Ac*. *polyacanthus* displayed 25 DEGs (Table S6) in the regulation of intracellular pH (pH_i_) (GO:0051453), including anion exchangers (AE1, AE2), vacuolar‐type H^+^ pumps (VHA: VPP1, VPP2, VATA), a monocarboxylate transporter (MCT), and electroneutral Na^+^/HCO_3_^−^ co‐transport (NBC) (Figure 3a). Only a few related genes with changes in expression were found for the other species (*D*. *aruanus*: CAH1; *P*. *moluccensis*: SL9A5; *O*. *compressus*: MYO1E; Table S6). These core pH_i_ regulation genes are involved in the extrusion of intracellular H^+^ or uptake of extracellular HCO_3_^−^ during acid–base regulation in many fishes (e.g., rainbow trout, dogfish, zebrafish (Brauner et al., 2019)). In addition, CAH1 and CAHZ are precursors to, or directly related to carbonic anhydrase (CA), the enzyme that catalyzes the rapid conversion of CO_2_ to H^+^ and HCO_3_^−^ and the reverse. CAH1 was upregulated in both *Ac*. *polyacanthus* and *D*. *aruanus* from the CO_2_ seep. This pattern has been found in other fishes, albeit upon exposure to extremely high CO_2_ levels (Georgalis et al., 2006; Perry et al., 2010). However, the CA‐dependent mechanism may be in place during a generalized acidosis—one that would ensue with exposure to elevated environmental CO_2_—to maintain or even enhance O_2_ delivery to tissues (Rummer & Brauner, 2011; Rummer et al., 2013). Furthermore, cardiac β1‐adrenergic receptors (ADRB1), which are part of the stress response and could help relieve impairments from potential hypercapnic tachycardia (Miller et al., 2014), were significantly expressed in *Ac*. *polyacanthus* from the CO_2_ seep. Likewise, hemoglobin (HBA, HBA1, HBB), which is the primary protein responsible for O_2_ transport (Rummer & Brauner, 2015; Rummer et al., 2013), was also significantly expressed at elevated levels in *Ac*. *polyacanthus* from the CO_2_ seep. Because a generalized acidosis reduces the affinity and carrying capacity of hemoglobin for O_2_, increased expression of various hemoglobin genes as well as those related with CA, as mentioned above, could counter the effect of a generalized acidosis such that O_2_ uptake and delivery are safeguarded (Harter & Brauner, 2017; Munday et al., 2019b; Rummer & Brauner, 2011). What is interesting is that these responses related to acid‐base balance and O_2_ delivery were observed exclusively in *Ac*. *polyacanthus*. As such, *Ac*. *polyacanthus* is sensitive to elevated *p*CO_2,_ with regulations to pH_i_ that may provide this species advantages to cope with elevated *p*CO_2_. In fact, while other pathways found here have been previously reported in *Ac*. *polyacanthus* in controlled laboratory studies (Schunter et al., 2016, 2018), the adjustments related to intracellular pH have not previously been shown, which reveals a key mechanism when fish are exposed to elevated *p*CO_2_ in the wild.

Neural signal transductions are mediated through ion channels and pumps to passively or actively push ions in and out of cells (Gadsby, 2009). *Ac*. *polyacanthus* sampled from the CO_2_ seep exhibited significant expression changes in a larger number of ion transporters compared with individuals from the control reef (Figure 3b; Figure S4, Table S7), a pattern not found for other species (Figure 3b; Figure S4). Interestingly, *Ac*. *polyacanthus* from the CO_2_ seep displayed overall repressed expression for Ca^2+^/K^+^ transporters (Table S7). Of these transporters, voltage‐dependent calcium channels and KCC2 are involved in the GABAergic pathway. This pattern, along with the attenuated expression of adenylyl cyclase (ADCY2), one GABA_A_ receptor (GBRB3), and two GABA_B_ receptors (GABR1, GABR2), implicates that *Ac*. *polyacanthus* displayed a repression of the GABAergic pathway when exposed to elevated *p*CO_2_ (Table S8, Figure S5). Besides, elevated *p*CO_2_ induced *Ac*. *polyacanthus* with a decreased expression in genes related to neurotransmitter signals, such as five evolutionarily conserved protein families (RIMS, UN13b, RIMB2, LIPA2, RB6I2) (Sudhof, 2012) and two glutamate receptors (NMDE4, NMDE3A) (Brassai et al., 2015). Hence, *Ac*. *polyacanthus*, when faced with long‐term elevated *p*CO_2_ in the wild, use a large repertoire of neuronal regulators that potentially affect neural signal transduction in synapses.

The ion and neuronal regulatory activity in *Ac*. *polyacanthus* might display rhythmic expression patterns driven by the CCGs. Among these ion transporters with differential expression exposed to elevated *p*CO_2_, KCNC1, KCMA1, HCN2, pH_i_ regulation genes (MCT2, AE2, VPP1, VPP2, NBC), and GABAergic pathway genes (voltage‐dependent calcium channels, KCC2) have been previously demonstrated in mammals (Aguilar‐Roblero et al., 1993; Colwell, 2011; Mure et al., 2018). Moreover, GABA receptors were also reported with rhythmic expression in mammalian brains (Aguilar‐Roblero et al., 1993; Colwell, 2011). Thus, transcriptional changes in CCGs may allow *Ac*. *polyacanthus* to flexibly adjust the rhythmic gene activity in the response to elevated *p*CO_2_.

### 2.3 Plasticity in transcriptional changes of *Acanthochromis polyacanthus* at the CO_2_ seep

**FIGURE 4:**
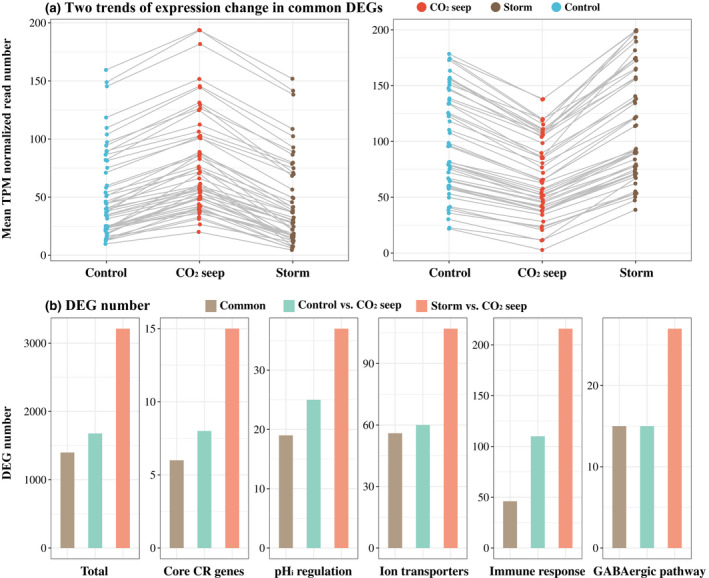
Over‐compensated expression patterns to acute environmental changes during a storm in *Acanthochromis polyacanthus* individuals at the CO_2_ seep. (a) Two trends of expression changes are detected in 1399 common differentially expressed genes (DEGs) between storm versus CO_2_ seep individuals, and control versus CO_2_ seep individuals. For illustration purposes, a subset (right: 52 genes; left: 51genes) of these common DEGs with normalized reads counts <= 200 were selected. Gene expression increased (left) or decreased (right) from control reef to CO_2_ seep but over‐compensated from CO_2_ seep to storm by a downregulation (left) or upregulation (right). (b) The number of DEGs increased from CO_2_ seep to storm in core circadian rhythm genes, ion transporters, and those related to pH_i_ regulation, immune response and the GABAergic pathway

Flexibility in gene expression adjustments for *Ac*. *polyacanthus* was observed in the response to short‐term exposure to ambient *p*CO_2_ levels at the seep in our study. While our samples were collected under stable environmental conditions (i.e., flat sea, little to no wind), a storm event lasting around 24 h provided the opportunistic collection of *Ac*. *polyacanthus* from the CO_2_ seep (hereafter, “storm” individuals). The wind during storm events, which by itself can decrease surface ocean *p*CO_2_ by 150–200 µatm (Massaro et al., 2012), can flush the oceanic ambient *p*CO_2_ waters into the CO_2_ seep, leading to a dilution of the high *p*CO_2_ waters. Hence, this storm event allowed us to also investigate the effects of a short‐term reduction in *p*CO_2_ on fish that have been exposed to chronic high *p*CO_2_ in the wild. Storm individuals showed a similar expression pattern to control reef individuals (97 DEGs; Table S1), but larger differences compared with CO_2_ seep individuals (3212 DEGs, Table S1; Figure S6). A common 1399 genes (Table S3) were differentially expressed between control reef and storm individuals compared with CO_2_ seep individuals (Figure S6). Moreover, if some genes of individuals from the CO_2_ seep exhibited upregulation, then the storm individuals had these genes downregulated to a lower expression in comparison with control and vice versa (Figure 4a), suggesting an “over‐compensated” pattern.80.3% of these common DEGs, such as the CCGs (Figure S7), revealed this “over‐compensated” pattern, which was also indicated from the increased number of DEGs enriched in a wide range of functions during the storm (Figure S8), such as the CR, pH_i_ regulation, ion transport, immune responses, and GABAergic pathways (Tables S4–S8; Figure 4b). The GABAergic pathway showed decreased expression in individuals from the CO_2_ seep compared with individuals from the control reef, but elevated expression during the storm (Figure S5). Hence, with high *p*CO_2_ water from the seep becoming mixed and replaced by ambient *p*CO_2_ oceanic water during the storm for 24 h, molecular adjustments in *Ac*. *polyacanthus* exposed to chronically elevated *p*CO_2_ were counteracted or even over‐compensated after acute *p*CO_2_ reduction. This species has been found in previous studies to be potentially adapted to fluctuating CO_2_, as they display better swimming performance and a greater aerobic scope than individuals from stable elevated *p*CO_2_ conditions (Hannan et al., 2020), together reflecting that *Ac*. *polyacanthus* can flexibly cope with variations *p*CO_2_. In addition, *Ac*. *polyacanthus* has been shown to exhibit larger molecular responses on short‐term exposure to elevated *p*CO_2_ (4 days) compared with chronic exposure (Schunter et al., 2018; Tsang et al., 2020). As such, the present study also reveals that acute changes in CO_2_ levels can cause larger transcriptional changes in the brain compared with chronic exposure to elevated *p*CO_2_ in the wild.

### 2.4 Disparity between species: Evolutionary rate and positive selection

**FIGURE 5:**
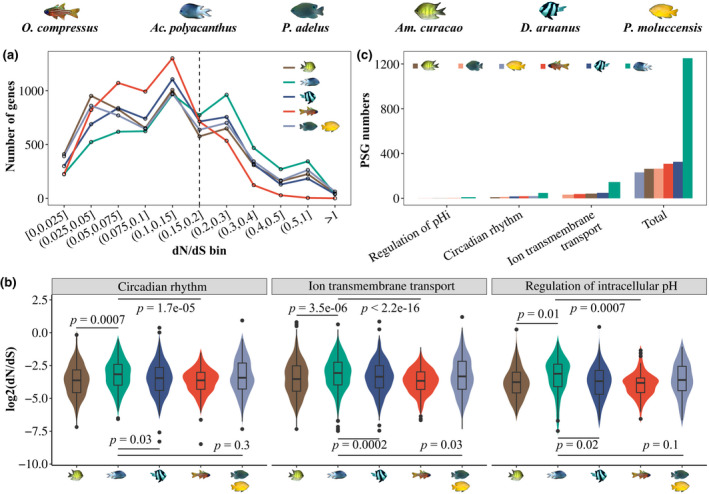
Evolutionary rate of five lineages (*Acanthochromis polyacanthus*, *Amblyglyphidodon curacao*, *Dascyllus aruanus*, *Ostohinchus compressus*, the most recent common ancestor of *Pomacentrus adelus* and *Pomacentrus moluccensis* (due to low qualified gene numbers for the two species)) and positively selected genes (PSGs) of all six species. (a) Number of genes within given bins of *d*_N_/*d*_S_ ratios with *Ac*. *polyacanthus* revealing more genes with elevated *d*_N_/*d*_S_ (> 0.15). (b) Rapid evolutionary rate of ion transmembrane transport, circadian rhythm, and regulation of pH_i_ in *Ac*. *polyacanthus*. (c) Number of PSGs in each species for three relevant functional categories and the total. *Ac*. *polyacanthus* displayed more PSGs than all of the other five species

Changes in gene expression are expected to drive evolutionary differences across species (King & Wilson, 1975; Wray, 2007), yielding divergent acclimation or adaptation potential to elevated *p*CO_2_. By applying Expression Variance and Evolution (EVE) models (Rohlfs & Nielsen, 2015) to the phylogeny of the six species (Figure S9), 183 genes were detected to have significantly diverged expression patterns among all species (Table S9). Of these, ATP7A and APBA2, two genes that are both involved in neural signal transduction, showed notable divergences between *Ac*. *polyacanthus* and the other species (Figure S10). Evolutionary differences are also partly driven by protein sequence divergences across species (King & Wilson, 1975; Wray, 2007). Hence, based on the phylogenetic tree (Figure S9), we used nonsynonymous and synonymous ratios (*d*_N_/*d*_S_) as indicators of protein sequence divergences to estimate gene evolutionary rate and positive selection for each species. *Ac*. *polyacanthus* exhibited a more rapid evolutionary rate than other species (Figure 5a, Table S10). Moreover, *Ac*. *polyacanthus* has a more rapid evolutionary rate also in genes related to the CR (GO:0007623), regulation of pH_i_ (GO:0051453), and ion transmembrane transport (GO:0034220; Figure 5b, Table S11), which also exhibit large expression differences between the CO_2_ seep and control site. This species, therefore, displays a genetic adaptive system with evolved expression patterns in key functions to cope with elevated *p*CO_2_.

Positive selection is an important source of evolutionary innovation and a major force behind the divergence of species (Kosiol et al., 2008) and can, therefore, reveal adaptive potential. By the comparisons between protein sequences of all six species in this study, *Ac*. *polyacanthus* displayed more positively selected genes (PSGs) (1251 PSGs, Table S12) with non‐synonymous mutations in comparison with the other species in general (Figure 5c, Table S13) but also in genes related to the CR (48 PSGs), regulation of pH_i_ (10 PSGs), and ion transmembrane transport (145 PSGs). An interaction of these PSGs with the DEGs may then facilitate gene expression changes in response to elevated *p*CO_2_ (Figure S11). For instance, 51.9% of interactions in the CR network for *Ac*. *polyacanthus* (95 genes: 28 PSGs, 64 DEGs, 3 were both PSG and DEG; 314 interactions) were differentially expressed and exhibited positive selection (Figure 5d, Figure S12). The most notable gene in this network was cyclic AMP‐responsive element‐binding protein 1 (CREB1) with four sequential nonsynonymous mutations at the binding domain (Figure S13). CREB1 is a well‐known transcription factor that drives the transcription and translation of clock genes (Colwell, 2011; O'Neill et al., 2008), which interact with DEGs including CCGs (BMAL1, CRY1, PER1, NFIL3), an ion transporter gene (KCMA1), and GABAergic pathway genes (ADCY2, ADCY9; Figure S12). Due to this, the circadian output of *Ac*. *polyacanthus* exposed to *p*CO_2_ is promoted by the PSGs. Compared with other species, *Ac*. *polyacanthus* may also have experienced an increased and higher evolutionary rate in gene expression over the past 15 million years, which can be seen in DEGs related to pHi regulation (CAHZ, B3AT, VPP1, S4A8; Figure S14a), CR (BMAL1, RORB, CLOCK, CREB1; Figure S14b), ion transport (CAC1A, CAC1C, KCNC1, KCNA3; Figure S14c), and GABA receptors (GABA_B_: GABR2; GABA_A_: GBRB1, GBRB3, GBRA3; Figure S14d). Genes with rapid evolutionary rate may, therefore, be the key in providing *Ac*. *polyacanthus* with more transcriptional plasticity, also the species with the largest transcriptional response in the wild under thermal stress (Bernal et al., 2020).

In fact, *Ac*. *polyacanthus* is known to have strong population genetic structure and even possible divergence within the species (Miller‐Sims et al., 2008; Planeset al., 2001) possibly owing to the direct development of juveniles and lack of a larval dispersal phase. While we can only hypothesize, it is possible that this life‐history trait leaves *Ac*. *polyacanthus* with enhanced potential to adapt to local conditions more than species with a dispersive larval phase and as a species therefore experiences elevated evolutionary rates and positive selection, which in turn provides this species with more transcriptional plasticity to respond to environmental changes. In addition to low evolutionary rate, the other fish species with higher dispersal may cope with environmental changes more frequently than *Ac*. *polyacanthus*, therefore these species are not as sensitive to environmental changes as *Ac*. *polyacanthus* and exhibited lower transcriptional plasticity in response to elevated *p*CO_2_.

Environmental conditions, including *p*CO_2_ levels, can change rapidly in nature, which is not often recreated under laboratory conditions. Here, we show that a tropical fish species, *Ac*. *polyacanthus*, displayed a means to react to elevated CO_2,_ as well as variability in environmental CO_2_ at a natural CO_2_ seep, through high transcriptional plasticity. Wild populations of six coral reef fish species revealed gene expression changes in common elements, in particular CR and immune responses. Notably, CCG expression changes of *Ac*. *polyacanthus* cause distinct downstream effects on pH_i_ regulation, ion transporters, and GABAergic pathway, which may promote the modulation of acid–base balance and neural activity under elevated *p*CO_2_ conditions in this species. Its expression changes due to chronically elevated *p*CO_2_ were rapidly compensated by an acute *p*CO_2_ reduction. Moreover, *Ac*. *polyacanthus* exhibited an overall rapid evolutionary rate and more positive selection, which may facilitate the transcriptional changes in elevated *p*CO_2_. Our work uncovered that some wild coral reef fish species possess a plastic molecular toolkit for the acclimation as well as potential adaptation to future OA conditions.

## 3 MATERIALS AND METHODS

### 3.1 Sampling and sample processing

Adult coral reef fishes were collected at a shallow coral reef CO_2_ seep at Upa Upasina in Papua New Guinea's Milne Bay Province (9^o^49.45′S, 150^o^49.07′E) and at the adjacent reef that is not affected by elevated *p*CO_2_ but is approximately 500 m away from the CO_2_ seep (see Fabricius et al. (2011)) for more details of the location and seawater chemistry parameters) in June 2018. Nearly all (>99%) of the volcanic gases at the Upa Upasina seep were CO_2_. The median pH and *p*CO_2_ of seawater at seeps were 7.77 units and 843 µatm, comparing with a higher pH (8.01 units) and lower *p*CO_2_ (443 µatm) at control sites. There are no significant difference of the temperature and salinity between the CO_2_ seeps and control sites (Fabricius et al., 2011). This study was conducted in accordance with James Cook University animal ethics permit A2534. Six selected fish species (130 individuals) were sampled across multiple days, with each species being collected from the CO_2_ seep and control reefs at the same time of day (Table S14). The six species were selected due to their abundance at the control reef and CO_2_ seep and include five damselfishes, *Ac*. *polyacanthus* (control: 11, CO_2_ seep: 7; storm: 9), *Am*. *curacao* (control: 9, CO_2_ seep: 10), *D*. *aruanus* (control: 10, CO_2_ seep: 10), *Pomacentrus adelus* (control: 11, CO_2_ seep: 11), and *Pomacentrus moluccensis* (control: 11, CO_2_ seep: 12), and one cardinalfish, *O*. *compressus* (control: 10, CO_2_ seep: 9). Specimens were caught alive underwater with barrier nets and/or clove oil by SCUBA divers and immediately brought to the boat by breath hold divers (snorkelers) for immediate dissection. Fish were sacrificed by a cut to the spinal cord, measured for body length, and whole brain tissue was dissected out and immediately placed in RNAlater. The samples were frozen after 24 h and placed in −80℃ on return to the laboratory. Whole brain tissue was removed from the freezer for processing in the molecular laboratory and extraction of RNA. RNAeasy kits (Quiagen) were used according to the manufacturer's protocol after samples were homogenized with sterile on‐use silicon beads in a beat beater (Fisherbrand) for 30 s. RNA quantity and quality were evaluated on a nanodrop, and Agilent Bioanalyzer and RIN values above 8 were accepted. RNA sequencing libraries were produced with Illumina TruSeq Kits v3 and sequenced for paired end 150b reads on 8 lanes of Hiseq4000 (the average read number is 37.8 million; Table S15) at the Bioscience core lab at the King Abdullah University of Science and Technology.

### 3.2 Sequence processing and transcriptome assembly

To only work with high‐quality sequences we assessed the raw read quality with FastQC v0.11.8 by removing adapter sequences and low‐quality sections and reads with Trimmomatic v0.30 (ILLUMINACLIP: TruSeq3‐PE‐2.fa:2:30:10 LEADING:4 TRAILING:3 SLIDINGWINDOW:4:20 MINLEN:40(Bolger et al., 2014), which resulted in the removal of 2.5% of all reads (Table S15). To address potential contaminates from the sequence reads we removed potential archaea, bacteria, fungi, and viral reads with Kraken v2.0.7‐beta (Wood & Salzberg, 2014) (‐‐confidence 0.7), which filtered out around 5 million reads (Table S15). For our six sampled species, only *Ac*. *polyacanthus* has an available reference genome (ENSEMBL ASM210954v1). We used this reference to map the high‐quality reads, whereas for the other five species (*Am*. *curacao*, *O*. *compressus*, *D*. *aruanus*, *P*. *adelus*, *P*. *moluccensis*) *de novo* transcriptomes needed to be assembled. The de novo assemblies were performed using DRAP v.1.92 (Cabau et al., 2017), a pipeline that includes many software packages (e.g., Trinity, Oases, CD‐HIT and TransDecoder) to produce corrected and nonredundant sets of transcripts per species. On average 603 million high‐quality reads were used to create species‐specific transcriptome references. The assemblies with DRAP (all_contigs.second_pass.fa) resulted in 397,121 contigs for *Am*. *curacao*, 412,639 for *O*. *compressus*, 380,320 for *D*. *aruanus*, 479,525 for *P*. *adelus*, and 639,615 for *P*. *moluccensis*. Among these contigs, average 210,701 open reading frames (ORFs), of which average 134,047 were complete, were detected by TransDecoder v3.0.1(Haas & Papanicolaou, 2018). The final set of transcripts per species only included the transcripts with ORFs. To estimate the quality of our de novo assemblies, ORFs and orthologous genes, BUSCO v2.0 (Simao et al., 2015) and Transrate v1.0.3 (Smith‐Unna et al., 2016) were applied to assess their completeness and quality scores, which indicated that they include almost single copy orthologues in the Actinopterygii database (Table S16), and good contig ratio in orthologous genes were much higher (82.9%–93.8%) than de novo assemblies and ORFs (Table S17).

### 3.3 Orthologous gene detection and annotation

To be able to compare the molecular response across the six species, we detected orthologous genes based on the ORF protein sequences using the default parameters in OrthoFinder v2.3.3 (Emms & Kelly, 2015). 116,503 initial orthologous groups among the six species were then filtered to contain at least one transcript for each species (remaining 17,564 orthogroups). We further filtered out groups based on alignment quality and number of mapped reads. Firstly, nucleotide sequences of the ORFs in each species were concatenated as the raw references to estimate the initial number of mapped reads for each transcript by RSEM v1.3.2 (Li & Dewey, 2011). Secondly, all transcript protein sequences in each group were then mapped to the Swiss‐Prot database (560,823 proteins, release‐2019–11) in UniProt (release‐2019–11) by BLASTP (BLAST v2.6.0+) with the default parameters. Secondly, for each transcript, only the top 10 hits (based on E‐value) with E‐value <1E‐25 were selected. If a transcript was blasted to different positions of the same protein, the transcript with the smallest E‐value was kept. Thirdly, For BLAST hits per species that were assigned to the same protein, we selected the one with highest number of mapped reads for each species. Finally, we removed the orthogroups with blast hits to the same protein in all six species if their average E‐value of the six hits was larger than 1E‐30. When more than one protein was detected in the same orthogroup, the one with largest total bit score was retained. As a result, 14,635 orthologous groups (Table S18) that include only one representative transcript per species were chosen as the final set of orthologous genes for the further analysis. The final set of orthogroups were then annotated using the UniProt Blast results that represent each orthologous group and GO terms and InterPro pathways were added with OmicsBox (Conesa et al., 2005).

### 3.4 Gene expression analyses

To identify gene expression differences between samples collected at CO_2_ seep and control reefs, the high‐quality reads were mapped against the reference transcript for each species with Bowtie 2 v2.3.5.1 (Langmead et al., 2009) by the default parameters. RSEM v1.3.2 (Li & Dewey, 2011) was then used with default parameters to generate transcript read number matrices. To statistically detect the DEGs between samples from CO_2_ seep and control reefs, raw read matrices were input into DESeq2 (Love et al., 2014) and first analyzed with a likelihood ratio test (LRT). After the removal of individuals showing very different gene expression with other individuals of the same species, genes were accepted as differentially expressed with an FDR adjusted P‐value ≤0.05 and the average of the normalized count values (basemean) ≥10 as well as the effect size (Log2FoldChange) ≥0.3. Gene expression patterns were evaluated across all species, which exhibited species‐specific brain gene expression profiles, independent of CO_2_ exposure (Figure S15). Functional enrichment analyses were performed for all gene sets of interest by comparison with the whole gene data set with a Fisher's exact test in OmicsBox. Functions were accepted as significantly enriched with a false discovery rate (FDR) Padj <0.05 and reduced to most specific terms. Using the significantly enriched GO terms in *O*. *compressus* with name including “B cell,” “T cell,” “T‐helper” (Table S6) as immune‐related functions, all DEGs of each species underlying these functions were extracted as the DEGs related to immune functions. And the genes with name including “calcium”, “sodium”, “potassium”, and “chloride” were regarded as the preliminary ion transport–related genes.

To understand gene expression shifts in the six species through a phylogenetic perspective, the analysis of EVE model (Rohlfs & Nielsen, 2015), which models gene expression as a quantitative trait across a phylogeny to estimate expression variance within versus among species, was performed based on a maximum likelihood (ML) tree of the six species. To prepare the ML tree for EVE model, the species‐specific protein sequence in each orthologous group were aligned, respectively, using Clustal Omega v1.2.4 (Sievers et al., 2011) (‐t Protein), and the gaps within alignments were removed by Gblocks v0.91b (Talavera & Castresana, 2007) (‐t=p). The orthologous group would be excluded when any sequences was shorter than 50 codons, which left 14,456 of the total orthologous groups. To construct the ML tree RAxML‐NG v0.9.0git (Kozlov et al., 2019) was run based on concatenate sequences (7,096,195 base pairs, 83.61% invariant sites) of the orthologous groups. The best ML tree was selected based on 50 random and 50 parsimony‐based starting tree topologies and the LG +G4m model (LG substitution matrix with discrete GAMMA N categories, ML estimate of alpha) (Bernal et al., 2020). The 1% threshold of nonparametric bootstrapping was reached at 50 iterations. Using scripts wrapped in RSEM v1.3.2(Li & Dewey, 2011), all orthologous genes expression levels were normalized for sequencing depth and transcript length by transforming to transcripts per million (TPM) by “abundance_estimates_to_matrix.pl”. And then the TPM values were normalized cross‐sample by “run_TMM_scale_matrix.pl” and then square root transformed. The best ML tree and normalized TPM values of reads number (from DEseq2, 14,635 orthologous genes) of samples from CO_2_ seep and control reefs were applied to run the EVE model, respectively. Significant genes with FDR *p* < .05 were selected as the lineage‐specific divergence genes (higher differences between lineages) and plastic genes (higher differences in expression within their own lineage than between lineages).

### 3.5 SNP calling, population structure, and outlier loci detection

To identify whether the potential genetic variances exist between samples from CO_2_ seep and control reefs, SNP calling was performed among all six species. Samples were individually mapped using Bowtie2 against the references (as described above). The reads per sample were sorted, assigned to new read‐group, marked duplicate, and reordered by PICARD in the GENOME ANALYSIS TOOLKIT (GATK v3.8–1–0) (DePristo et al., 2011), which was used for SNP calling and SNP filtering (Figure S1c). After splitting the reads that contain Ns in their cigar string, the SNPs within samples were detected using HaplotypeCaller in GATK. At first, the genotypes were filtered based on the Phred‐scaled quality score using a threshold of 30. All resulting SNPs were merged together by “‐T CombineVariants ‐genotypeMergeOptions UNIQUIFY.” Next, potential SNPs were removed if there were more than three SNPs in 35 base pair window size. We also filtered out potential SNPs if any of the following conditions occurred: the confidence/quality by depth was <5.0, read depth was <10, z‐score from Wilcoxon rank sum test of Alt versus ref read position bias was < −8, z‐score from Wilcoxon rank sum test of Alt versus Ref read mapping quality was < −12.5, root mean square (RMS) of the mapping quality of reads across all samples was <40, phred‐scaled p‐value using Fisher's exact test was >60, or more than 10% of the reads had a mapping quality of zero. Furthermore, only the SNPs with “PASS” flag, heterozygosity <80% (to remove potential paralogs) and minor allele frequency (MAF) ≥0.1 (to exclude recent mutations that are uninformative loci) (Berdan et al., 2015) were retained. To compare the sequence variance between individuals from CO_2_ seep and control site, VCFtools (v0.1.13) was used to keep biallelic SNPs with “PASS” flag and remove SNPs with low F_ST_ (0 or “non”), low MAF (< 0.1, to exclude recent mutations that are uninformative loci) (Roesti et al., 2012) and high heterozygosity (large than 0.8, to remove potential paralogs) (Berdan et al., 2015). Once the high‐quality SNPs were obtained between samples from CO_2_ seep and control site, ADMIXTURE v1.3.0 (Alexander & Lange, 2011) was performed to estimate individual ancestry from the SNPs data sets by simulated K (the priori number of groups) from 1 to 10. Putative outlier loci were detected using a Bayesian approach implemented in BayeScan 2.0 (Foll & Gaggiotti, 2008) under two different assumptions that the samples from CO_2_ seep and control site were one population or two different populations.

### 3.6 Estimation of the evolutionary rate and positively selected genes

The ML tree was also applied to estimate the evolutionary rate of our six species. To estimate lineage‐specific evolutionary rates and positively selected genes (PSGs), the nucleotide sequence of each orthologous gene per species were aligned to the representative protein by GeneWise (Birney & Durbin, 2000) (‐quiet ‐genesf ‐pseudo ‐both) using the UniProt protein sequence of each orthologous group as the representative protein. After the alignments obtained by Clustal Omega v1.2.4 (Sievers et al., 2011) (‐t DNA), Gblocks v0.91b (Talavera & Castresana, 2007) (‐t=c) was used to keep the conserved regions and remove the gaps in alignments of all species for all orthologous genes. Only the final sequences with length more than 150 would be considered in the following analysis, which left 12,829 orthologous groups.

To estimate the lineage‐specific evolutionary information, the free‐ratio model of codeml in Phylogenetic Analysis by Maximum Likelihood (PAML) (Yang, 1997) was run on each orthogroup. The results for genes of each lineage under study were curated to reduce errors (Goodman et al., 2009) by the removal of genes with any one of the following values: *d*_S_ >1, N > sequence length, N + S > sequence length by 50 or more bp, and N*d_N_ or S*d_S_ <1. The final gene number for the evolutionary estimation were 10,535 (*Ac*. *polyacanthus*), 10,890 (*D*. *aruanus*), 11,174 (*O*. *compressus*), 8444 (*Am*. *curacao*), 4346 (*P*. *adelus*), and 4632 (*P*. *moluccensis*). Due to the low number of estimation for *P*. *adelus* and *P*. *moluccensis*, the *d*_N_/*d*_S_ of the most recent common ancestor (MRCA) of the two species (8750 genes) were extracted as their evolutionary rate. Finally, 5816 orthologous genes with curated *d*_N_/*d*_S_ in all five lineages (*Ac*. *polyacanthus*, *Am*. *curacao*, *D*. *aruanus*, *O*. *compressus*, the ancestral lineage of *P*. *adelus* and *P*. *moluccensis*), were used in the *d*_N_/*d*_S_ comparisons. Genes were then classified into dN/dS ratio bins (Figure 5a) for all of the five lineages. To compare the evolutionary rate of genes associated to certain functions between the five lineages, genes underlying each GO function were extracted and then their mean *d*_N_/*d*_S_ were compared by Wilcoxon signed‐rank test.

The branch‐site model of codeml in PAML was applied to investigate the PSGs. The terminal branch of each species was set as the foreground branch, and a Likelihood Ratio Test (LRT) was used to estimate whether the branch‐site model containing positively selected codons (omega >1) fits better than the null model including neutral selection or negative selection (omega ≤1). Chi‐square statistics wrapped in PAML were performed to estimate the significance of model comparisons, and the P values were FDR corrected using R version 3.6.3. Only the genes with an LRT FDR <0.05 and containing codon sites with a posterior probability of positive selection over 0.95 by the Bayes empirical Bayes method were treated as PSGs. To understand if elevated number of PSGs are associated with certain functions we also extracted the genes showing PSGs underlying these functions per species.

To verify the evolutionary rate dynamics among *Ac*. *polyacanthus* and other five fish species, the trait evolutionary rate across all six species were evaluated by Bayesian analysis of Macroevolutionary Mixture (BAMM) (Rabosky, 2014). The normalized reads number of orthologous genes across six fish species were used as the trait data. A phylogenetic tree that is time‐calibrated across all individuals of the six fish species is needed for BAMM. Firstly, we estimated the divergence time of the six fish species. Based on 2686 single‐copy orthologues across 12 fish species (the protein sequence of longest transcript per gene was selected, the 12 species were six fish species in our study, and Japanese Medaka, *Oryzias latipes*; Fugu, *Takifugu rubripes*; Stickleback, *Gasterosteus aculeatus*; Zebrafish, *Danio rerio*; Platyfish, *Xiphophorus maculatus*; Spotted gar, *Lepisosteus oculatus*) detected by OrthoFinder v2.3.3(Emms & Kelly, 2015). All orthologs were aligned using MUSCLE v.3.8.31(Edgar, 2004) with default parameters and the regions with bad quality were trimmed using trimAl v1.4.rev22 (Capella‐Gutiérrez et al., 2009) with “‐gt, 0.8; –st, 0.001; –cons, 60.” The resulting alignments were concatenated as input of RAxML v.8.2.11 (Stamatakis, 2006) for phylogenetic tree construction using the optimal amino acid substitution model searched by the parameter “PROTGAMMAAUTO”. We specified spotted gar as the outgroup and evaluated the robustness of the result using 100 bootstraps and then based on the resulting phylogenetic tree and the coding nucleotide sequences alignments of 2686 single‐copy orthologues across 12 fish species, MCMCTree (Yang, 1997) was used under a relaxed‐clock model with approximate likelihood calculation and ML estimation of branch lengths to infer divergence time with two fossil calibrations [*O*. *latipes*–*T*. *nigroviridis* (~96.9–150.9 million years ago (Mya)), *D*. *rerio*–*G*. *aculeatus* (~149.85–165.2 Mya)] (Du et al., 2020). The MCMC process was run for 5,000,000 steps and sampled every 5000 steps. MCMCtree suggested that the divergence time between *Ac*. *polyacanthus* and *Am*. *curacao* in our study is approximately 24.4 Mya (Figure S16), which is similar to previous estimates (McCord et al., 2021). Secondly, to construct the phylogenetic tree of all individuals in our study, we assembled the sequences of 50 randomly selected genes from 2686 single‐copy orthologues referring the methods in Kuang et al. (2018). We kept the longest ORFs estimated by TransDecoder v3.0.1(Haas & Papanicolaou, 2018) for each assembled gene, and the concatenated ORFs sequences (45,343 bp) of the final 31 single‐copy orthologs (Table S19) that were captured by all 130 individuals were aligned by MUSCLE v.3.8.31 (Edgar, 2004) and trimmed using trimAl v1.4.rev22 (Capella‐Gutiérrez et al., 2009) with “‐gt, 0.9; –st, 0.001; –cons, 60.” The resulting sequences were concatenated to construct the phylogenetic tree by RAxML v.8.2.11 (Stamatakis, 2006) using 1000 bootstraps under the general time reversible model as suggested by jmodeltest v.2.1.10 (Darriba et al., 2012). The phylogenetic tree of all 130 individuals (Figure S17) were used to obtain time‐calibrated tree by r8s v.1.81 (Sanderson, 2003) using the estimated age (approximately 24.4 Mya) between *Ac*. *polyacanthus* and *Am*. *curacao*. With the normalized reads count of genes as trait data, BAMM was run by modifying the control file with “modeltype = trait; expectedNumberOfShifts = 1.0; numberOfGenerations = 10000000; mcmcWriteFreq = 5000.” The convergence of MCMC chains was determined by plotting effective sample size of log‐likelihood and number of shifts in each sample.

### 3.7 Gene interaction network analysis

To further understand the interaction, and relative importance of DEGs and PSGs across six coral fish species, the gene interaction network was estimated using Cytoscape v3.7.0 (Shannon et al., 2003) to draw edges between genes with functional interactions reported for humans in the STRING database (Szklarczyk et al., 2016). Gene interaction network is selected if its nodes are more than three. The DEGs enriched in CR (GO:0007623) were extracted to understand the interactions between DEGs related to CR among *Ac*. *polyacanthus*, *D*. *aruanus*, and *P*. *moluccensis*. To understand the influences of PSGs on DEGs caused by elevated *p*CO_2_ in *Ac*. *polyacanthus*, the genes related to CR (GO:0007623), regulation of pH_i_ (GO:0051453), and ion transmembrane transport (GO:0034220) were extracted to build the gene interaction network. The betweenness centrality of node and edge were calculated by “Analyze Network” in Cytoscape. The interactions were classified by DEG‐DEG, PSG‐PSG, and DEG‐PSG to represent an interaction that only include DEGs or PSGs, or a DEG and a PSG.

## CONFLICT OF INTEREST

The authors declare no competing interests.

## AUTHOR CONTRIBUTIONS

P.L.M., J.L.R., T.R., and C.S. collected the samples with the support of R.R‐M. and I.N. C.S. prepared the samples for RNA sequencing. J.L.K processed the transcriptome data and with the help of C.S. analyzed and interpreted the data. J.L.K. and C.S. wrote the first draft, and all authors edited and approved the final manuscript.

## Supporting information

Supplementary Material

Table S1‐19

## Data Availability

RNA‐seq raw sequences and the de novo assembled transcriptome assemblies have been deposited in NCBI under BioProject PRJNA691990.
