# Supplementary material for: Rapid evolution fuels transcriptional plasticity to ocean acidification
Source: Glob Chang Biol. 2022 Mar 3;28(9):3007–22. doi: 10.1111/gcb.16119 (PMC9310587; doi:10.1111/gcb.16119)
Supplement: Supplementary file 1 — Supplementary Material [file GCB-28-3007-s001.docx]

**Rapid evolution fuels transcriptional plasticity to ocean acidification**

Jingliang Kang *^1^*, Ivan Nagelkerken *^2^*, Jodie L. Rummer *^3,4^*, Riccardo Rodolfo-Metalpa *^5^*, Philip P. Munday *^3^*, Timothy Ravasi ^3,^*^6^*^*^ and Celia Schunter^1*^

^1^Swire Institute of Marine Science, the division of Ecology and Biodiversity, The University of Hong Kong, Pokfulam, Hong Kong SAR

^2^Southern Seas Ecology Laboratories, School of Biological Sciences and The Environment Institute, DX 650 418, The University of Adelaide, Adelaide, SA 5005, Australia

^3^Australian Research Council Centre of Excellence for Coral Reef Studies, James Cook University, Townsville, Queensland, 4811, Australia

^4^College of Science and Engineering, James Cook University, Townsville, Queensland, 4811, Australia

^5^ UMR ENTROPIE (UR-IRD-IFREMER-CNRS-UNC), Centre IRD, BP A5, 98848 Nouméa cedex, New Caledonia

^6^Marine Climate Change Unit, Okinawa Institute of Science and Technology Graduate University, 1919–1 Tancha, Onna-son, Okinawa 904–0495, Japan

Supplementary Materials

Supplementary Figure 1:

**SNP analysis:**

9, 918, 136, 923, 13, 120 SNPs in *Am. curacao*, *Ac. polyacanthus*, *O. compressus*, *D. aruanus*, *P. adelus* and *P. molusscensis* among the individuals from the CO_2_ seep and Control sites. No outlier loci were detected among the individuals from CO_2_ seep and Control site for all six fish species. Excluding *Am. curacao*, all of the other five fish species showed the lowest cross-validation value when genetic structure equals 1, indicating there is no significantly genetic difference between fish in CO_2_ and Control in any species.

Figure S1a. Outlier SNP loci detection in all of the six coral fish species, which indicated no outlier loci (potential for selection) whether the individuals from CO_2_ seep and Control site.

Figure S1b. Genetic structure detection of six coral fish species. Excluding *Am. curacao*, all of other five species indicate the lowest value of cross-validation (CV) error when genetic structure number (K) equals to 1 (K=1 - 10).

Figure S1c. Diagram showing the pipeline used to call and filter SNPs for the detection of outlier loci and genetic structure using Bayescan and Admixture, respectively.

Supplementary Figure 2:


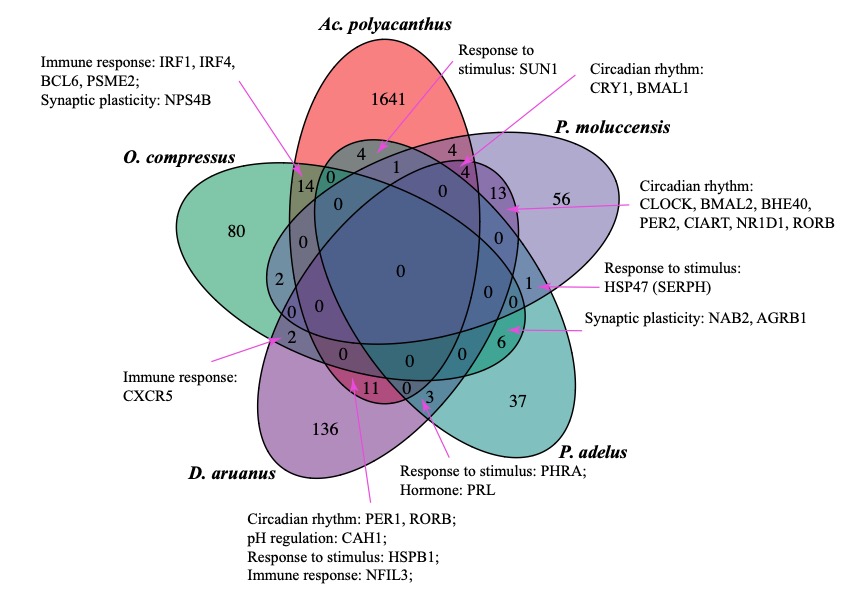


Figure S2. Venn diagram of differentially expressed genes (DEGs) in all species except *Am.curacao* (only one DEG not overlapped with any other species). These common DEGs indicate some consistent responses associated with response to stimulus, synaptic plasticity, circadian rhythm and immune response in the CO_2_ seep environment.

Supplementary Figure 3:

Number of differentially expressed genes (DEGs) in the GO term “circadian rhythm” (GO:0007623) used in the gene interaction network analysis: all uniprot ids were converted to human id in String database, from which to search the gene interaction and then select the main network (≥ 3 genes).

| Species | Total DEGs related to CR | String human id conversion | Total DEGs in the main network | Core CR DEGs in the main network | Other DEGs in the main network |
| --- | --- | --- | --- | --- | --- |
| *P. moluccensis* | 17 | 15 | 10 | 10 | 0 |
| *D. aruanus* | 32 | 29 | 15 | 15 | 0 |
| *Ac. polyacanthus* | 94 | 85 | 65 | 5 | 60 |


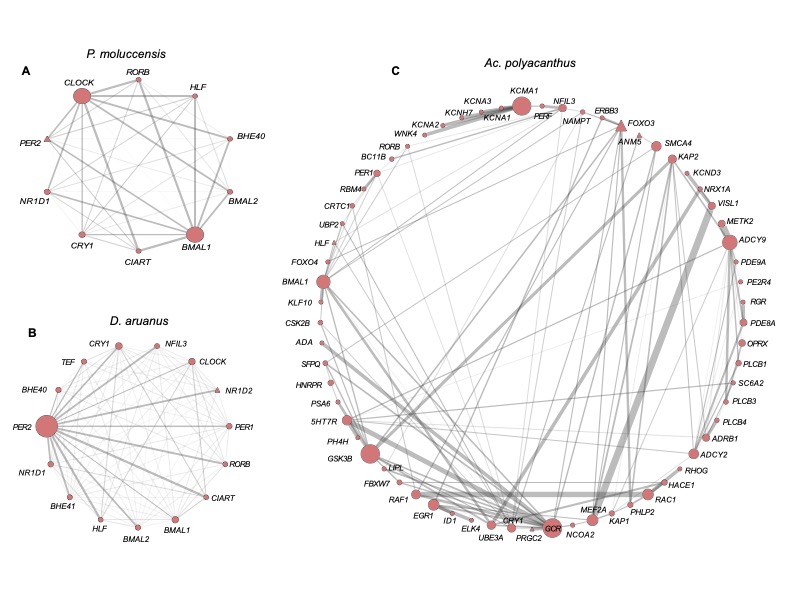


Figure S3. The main interaction network of DEGs in the GO term “circadian rhythm” (GO:0007623) in *P. moluccensis*, *D. aruanus*, and *Ac. polyacanthus*. ﻿Size of node (gene) represents its betweenness centrality, and width of edge between nodes represents the edge betweenness in the network. The triangle indicates that gene were under positive selection. All nodes in *P. moluccensis* (A) and *D. aruanus* (B) were core circadian genes. (C) For *Ac. polyacanthus*, in addition to the five core circadian genes (BMAL1, CRY1, PER1, HLF), there are other 60 DEGs in regulation of intracellular pH (UBE3A, HACE1, B3AT, B3A2), ion transmembrane transport (UBE3A, KCMA1, KCNA1, KCNA3, KCNA2, KCNH7, NRX1A, PLCB1, OPRX, MEF2A, WNK4, PLCB3), and GABAergic pathway (ADCY2, ADCY9).

Supplementary Figure 4:

Figure S4. Differentially expressed genes directly involved in the ion or ion-dependent transport in five fish species. Red indicates upregulation at the CO_2_ seep, and blue indicates downregulation. (A) Most genes herein were downregulated with elevated CO_2_ exposure in *Ac. polyacanthus*, in particularly for Ca^2+^/K^+^ related transporters. (B) The other four species (*Dascyllus aruanus*, *Pomacentrus adelus*, *P. moluccensis* and *Ostorhinchus compressus*) show much fewer transporter as compared to *Ac. polyacanthus*.

Supplementary Figure 5:

Figure S5. The regulation of genes in the GABAergic pathway in *Ac. polyacanthus*. (A) Overall repression of genes in the GABAergic pathway in *Ac. polyacanthus* exposed to elevated CO_2_. The only two upregulated genes are ADCY9 and GAT-2 which are responsible for the take-up of GABA in glial cells. (B) The upregulation of genes in storm samples as compared to CO_2_ seep. Storm samples reversed the transcriptional changes from elevated CO_2_ by reverse-regulating all of the differentially expressed genes between control and CO_2_ seep. In addition, storm samples also significantly increased expression of another twelve genes, including two GABA_A_ receptors (GABRA3, GABRB1) and ten other genes (PKA, PKC, 2*SRC, PLCL1, GAD, ABAT, ADCY1, ADCY5, ADCY8).

Supplementary Figure 6:


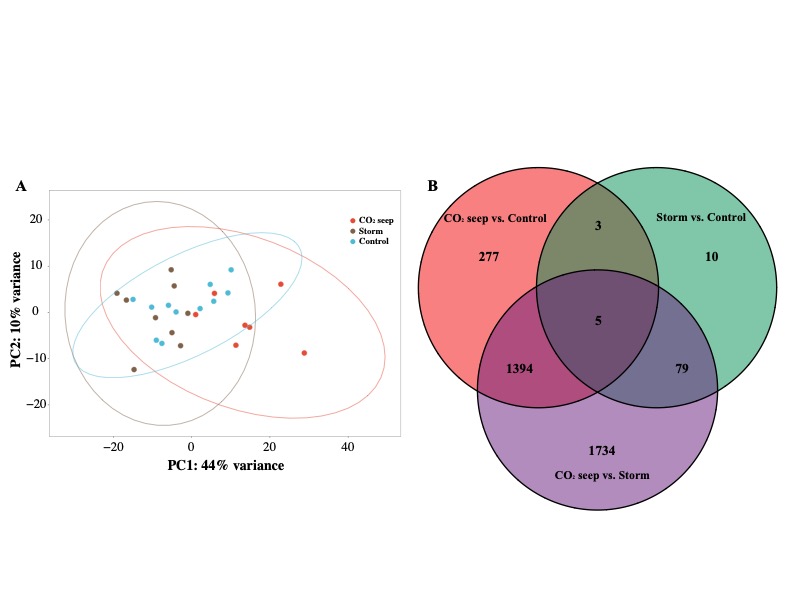


Figure S6. A similar expression pattern between storm and control individuals as compared to the CO_2_ seep individuals. (A) PCA based on gene expression of all *Ac. polyacanthus* individuals. Storm and Control individuals were clustered together. (B) Venn diagram of differentially expressed genes in *Ac. polyacanthus* between CO_2_ seep vs. control, CO_2_ seep vs. storm conditions, storm conditions vs. control. Storm samples showed differences with control but larger differences with CO_2_ seep.

Supplementary Figure 7:

Figure S7. The compensation or over-compensation seen of core circadian genes in the storm individuals. The star indicates genes between CO_2_ seep versus Control or CO_2_ seep vs. storm were significantly differential expressed.

Supplementary Figure 8:

Figure S8. The differentially expressed genes (DEGs) number involved in significant functions of seep vs. control and seep vs. storm. The X axis is the broad Gene Ontology functions with three points connected by a line representing a same GO term (common DEGs number between two comparisons (dark blue), DEGs numbers in seep vs. control (red), and DEGs numbers in seep vs. storm (light blue)). The boxes show functions only significant in seep vs. control, only significant in seep vs. storm, and significant in both two comparisons. Almost functions have large part of common DEGs (common DEGs numbers are nearly equal to the DEGs number in see vs. control) and DEGs number in seep vs. storm were always larger than seep vs. control (light blue point is always in the top of red point).

Supplementary Figure 9:

Figure S9. Phylogenetic tree used for the EVE and PAML analysis for the six collected coral reef fishes based on the protein sequences of 14,453 orthologous genes (Gblocks selects the conserved blocks, select the sequences longer than 50 codons, 7,096,195 bp, 83.61% invariant site, RAxML-NG select the best ML tree).

Supplementary Figure 10:

Figure S10. Significant genes with diverged expression profiles for the six coral fish species. Orthogroup expression levels were normalized for sequencing depth and transcript length by transforming to transcripts per million (TPM). The normalized reads number were used in EVE model analysis. The upper plot presents the likelihood ratio test value and adjusted P value of all orthologous genes, red point indicated the genes with a significant adjusted P value (< 0.05), and three genes (ATP7A, APBA2, FLRT2) showed the highest likelihood ratio test value, meaning their expression are prominent diverged between species. ﻿Barplot indicated two genes (ATP7A, APBA2) showed great divergence between *Ac. polyacanthus* and other species.

Supplementary Figure 11:

Figure S11. Proportion of the three interaction types in gene interaction network of PSGs and DEGs in *Ac. polyacanthus*. PSG-DEG, DEG-DEG, and PSG-PSG indicate the interactions between PSGs and DEGs, DEGs and DEGs, and PSGs and PSGs, respectively. PSG-DEG takes 51.9%, 47.1%, and 49.1% of all interactions in the whole interaction network of genes in circadian rhythm (95 genes, 314 interactions), regulation of pH_i_ (20 genes, 34 interactions), and ion transmembrane transport (276 genes, 1,086 interactions).

Supplementary Figure 12:


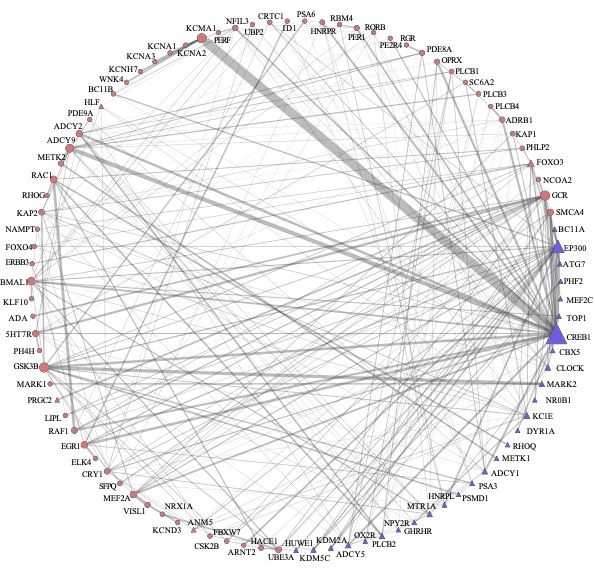


Figure S12. The gene interaction network of differential expressed genes (DEGs) and positively selected genes (PSGs) related to circadian rhythm in *Ac. polyacanthus*. ﻿Size of node (gene) represents its betweenness centrality, and width of edge between nodes represents the edge betweenness in the network. The triangle indicates PSGs, red indicates DEGs, and the red and triangle represent that genes were both PSGs and DEGs. The interaction network includes 98 genes (32 PSGs, 67 DEGs, 4 genes were both PSGs and DEGs) and 314 interactions, of which 163, 118, and 33 were PSG-DEG, DEG-DEG, and PSG-PSG, respectively.

Supplementary Figure 13:

Figure S13. Functional domain annotation of *Ac. polyacanthus* CREB1protein with online tool SMART^1^. *Ac. polyacanthus* showed a cluster of four nonsynonymous mutations (277 - 280) in the basic region leucin zipper (BRLZ) domain, which allows for both dimerization and sequence-specific DNA-binding interactions^2^.

Supplementary Figure 14:

Trends in evolutionary rates of gene expression across all six fish species in our study.


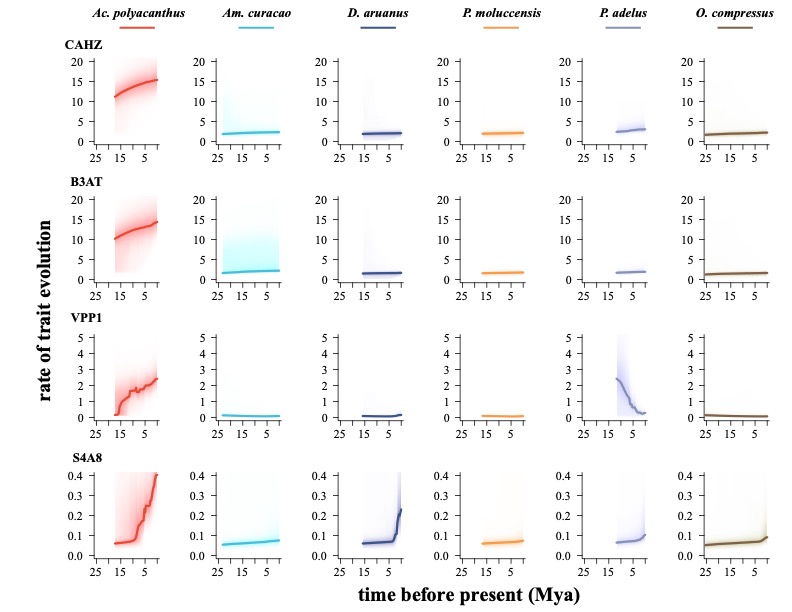


Figure S14a. Trends in evolutionary rates of expression of four genes (CAHZ, B3AT, VPP1, S4A8) related to intracellular pH regulation. Each row represents a gene and each column indicates a species (also displayed with different colors). Y-axis represents evolutionary rate of gene expression, and x-axis represents time before present (million years ago: Mya). The gradients represent confidence intervals for each of the estimated rates.


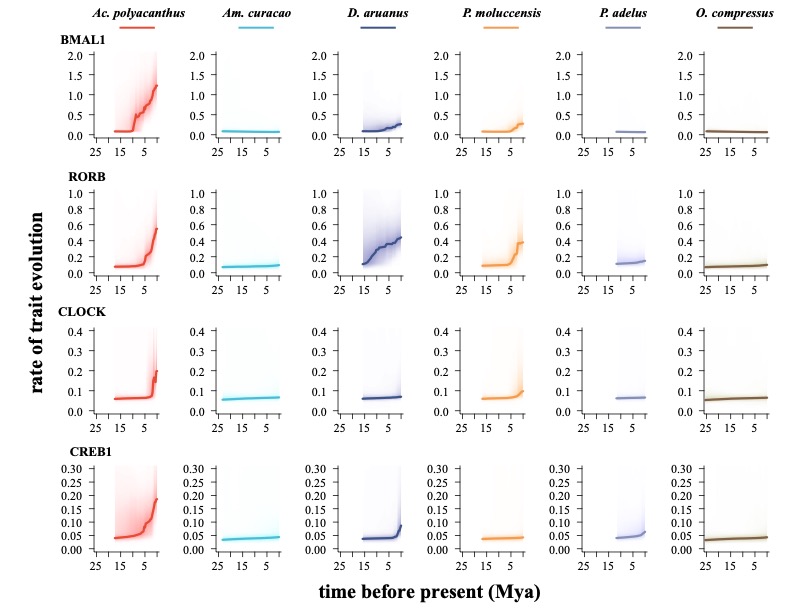


Figure S14b. Trends in evolutionary rates of expression of four genes related to circadian rhythm (BMAL1, RORB, CLOCK, CREB1) across six fish species. Each row represents a gene and each column indicates a species (also displayed with different colors). Y-axis represents evolutionary rate of gene expression, and x-axis represents time before present (million years ago: Mya). The gradients represent confidence intervals for each of the estimated rates.


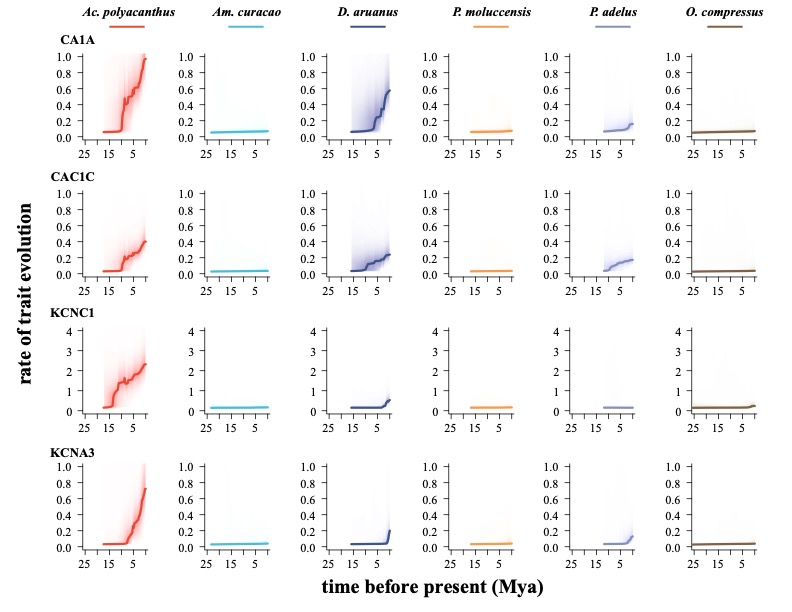


Figure S14c. Trends in evolutionary rates of expression of four genes related to ion transport (CAC1A, CAC1C, KCNC1, KCNA3) across six fish species. Each row represents a gene and each column indicates a species (also displayed with different colors). Y-axis represents evolutionary rate of gene expression, and x-axis represents time before present (million years ago: Mya). The gradients represent confidence intervals for each of the estimated rates.


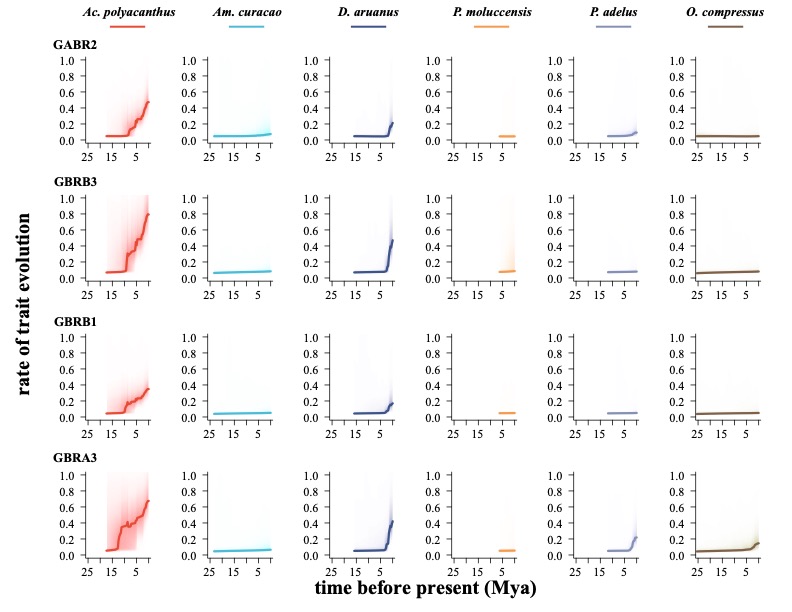


Figure S14d. Trends in evolutionary rates of expression of four GABA receptors (GABA_B_: GABR2; GABA_A_: GBRB1, GBRB3, GBRA3 across six fish species. Each row represents a gene and each column indicates a species (also displayed with different colors). Y-axis represents evolutionary rate of gene expression, and x-axis represents time before present (million years ago: Mya). The gradients represent confidence intervals for each of the estimated rates.

Supplementary Figure 15:

Figure S15. Clustering of fish individuals across six coral fish species by CO_2_ seep and control site based on the top 1000 variance genes. Fish species are indicated by different colours, individuals were clustered together according species, independent of individuals from CO_2_ seep or control sites.

Supplementary Figure 16:


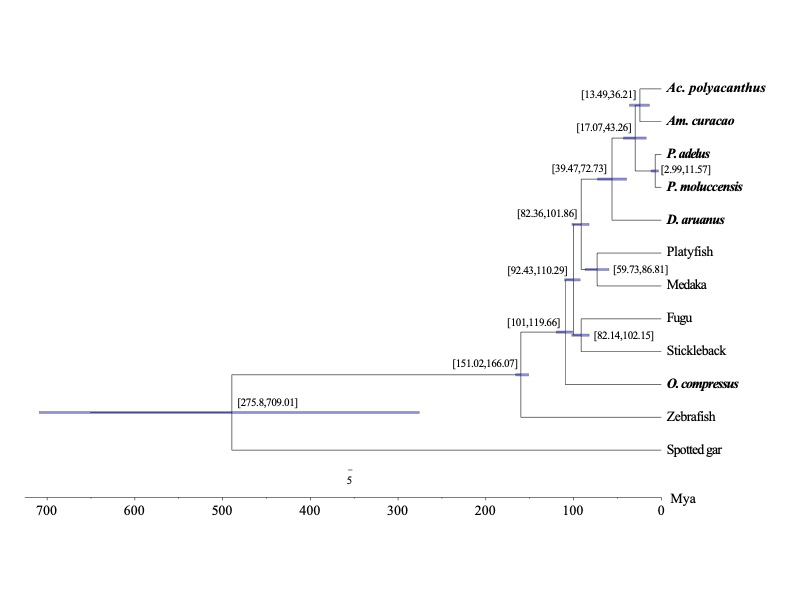


Figure S16. Divergence time of six coral fish species. The timescale was calculated from a phylogenetic tree based on 2,686 single copy orthologs across 12 fish species (six species in this study were bold) using MCMCtree. Branch lengths were calibrated by using the fossil records for the split of Medaka/Fugu and Zebrafish/Stickleback. Blue bars refer to the 95% confidence interval. Node labels represent the estimated time of node ages (95% confidence level). The posterior mean divergence time (about 24.4 Mya) between *Ac. polyacanthus* and *Am. curacao* would be applied to generate the time-calibrated phylogenetic tree for the BAMM analysis.

Supplementary Figure S17:

Figure S17. Phylogenetic tree of 130 individuals of six fish species based on the concatenated sequences of open reading frames of 31 single copy orthologs (45,343 bp in total). The phylogeny tree was constructed by RAxML under the general time reversible (GTR) model. The node with bootstrap support that equals to 100 was indicated as red circle in the middle of branch. This phylogenetic tree would be transformed to a time-calibrated phylogenetic tree for BAMM analysis based on the posterior mean divergence time (about 24.4 Mya) between *Ac. polyacanthus* and *Am. curacao*.

1 Letunic, I. & Bork, P. 20 years of the SMART protein domain annotation resource. *Nucleic acids research* **46**, D493-D496 (2018).

2 Ellenberger, T. Getting a grip on DNA recognition: structures of the basic region leucine zipper, and the basic region helix-loop-helix DNA-binding domains. *Current Opinion in Structural Biology* **4**, 12-21 (1994).
